# Spontaneous Coronary Artery Dissection: Case Series and Literature Review

**DOI:** 10.7759/cureus.13539

**Published:** 2021-02-24

**Authors:** Nouraldeen Manasrah, Ali F Al Sbihi, Kendall Bell, Luis C Afonso, Nimrod Blank

**Affiliations:** 1 Internal Medicine, Detroit Medical Center/Sinai Grace Hospital, Detroit, USA; 2 Internal Medicine, Wayne State University, Detroit, USA; 3 Medicine, Wayne State University, Detroit , USA; 4 Interventional Cardiology, Harper University Hospital, Detroit, USA

**Keywords:** spontaneous coronary artery dissection, myocardial infarction with no obstructive coronary atherosclerosis, postpartum myocardial infarction, intravascular ultrasound (ivus)

## Abstract

Spontaneous coronary artery dissection (SCAD) is an important and rare cause of myocardial infarction (MI), particularly among young women without traditional atherosclerotic risk factors. Late pregnancy and postpartum period are associated with more risk for developing SCAD. No enough data exist regarding the ideal management of SCAD due to lack of randomized trials comparing medical therapy and revascularization strategies.

We present three cases of SCAD, two of them were postpartum women while one involved an obese young woman with no identifiable risk factors. We describe the pathophysiology, types of SCAD, risk factors, clinical presentation, and management approach. This case series highlights the need to raise awareness of SCAD and to facilitate accurate diagnosis promptly.

## Introduction

Spontaneous coronary artery dissection (SCAD) is a nontraumatic and noniatrogenic separation of the coronary arterial wall and a rare cause of acute myocardial infarction (MI). SCAD is the most common cause of MI in pregnancy and postpartum period [1]. The management of SCAD differs from atherosclerosis-related acute coronary syndrome (ACS), and fraught with diagnostic challenges and significant therapeutic dilemmas due to the lack of evidence-based research [2]. Clinical outcomes are contingent on many factors like the site and extent of the lesion, number of affected vessels, and hemodynamic status.

## Case presentation

Case 1

A 32-year-old gravida 3, para 3 African American female with no past medical history had recently delivered a baby uneventfully. On postpartum day 9, she developed acute chest pain. It was pressure like, centrally located, aggravated with movement with no relieving factors. It was constant for a week. She went to a local hospital and was found to have elevated blood pressure (170/90 mmHg), but the rest of the vital signs were normal. Physical exam was unremarkable except for lower extremity edema that was improving since delivery. Labs showed mildly elevated troponin I (0.23 ng/mL normal range 0-0.4 ng/mL) and electrocardiogram (ECG) was normal. The rest of labs were unremarkable. Due to concern of ACS, left heart catheterization (LHC) was performed which showed normal coronary arteries. She was monitored in cardiac telemetry unit for two days then discharged home in a stable medical condition.

The patient reported that the pain did not resolve and was worsening after discharge, so she presented to the ED in our hospital after five days of her recent discharge. She described the chest pain as pressure like, centrally located, with radiation to both shoulders. She felt like her symptoms were worse with exertion and better with rest. Vital signs were stable on admission except for blood pressure of 168/88 mmHg. Physical examination was unremarkable.

Laboratory evaluations including complete blood count, complete metabolic panel, lipid profile, and coagulation profile were normal. Urine drug screen was negative. ECG showed inverted T waves in V4-V6. Echocardiogram showed reduced left ventricular ejection fraction of 35%-40%, severe hypokinesia in inferior-lateral wall and apex. No valvular abnormalities were observed.

**Figure 1:**
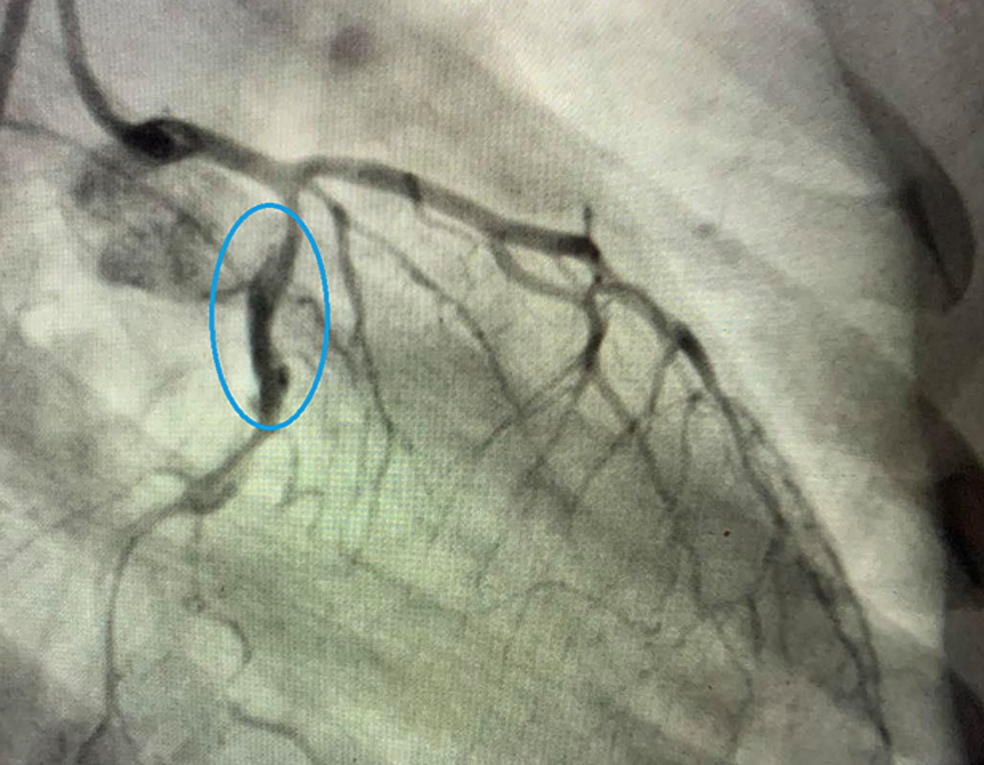
LHC shows dissection in mid to distal left anterior descending artery and circumflex artery. LHC, left heart catheterization

The patient was started on IV heparin infusion, aspirin, ticagrelor, and atorvastatin. Sublingual nitroglycerin was given. Emergent LHC was performed, which revealed extensive dissection in mid to distal left anterior descending and proximal to distal left circumflex coronary arteries as shown in Figure 1.

Heparin infusion was promptly stopped, and the patient was subsequently admitted to medical intensive care unit (MICU) for monitoring. Given her coronary anatomy with lack of “high risk features”, a decision was made to pursue medical management. She was treated with aspirin, beta blocker, and ACE-Inhibitor. She was monitored in the MICU for 48 hours. She was feeling better, transferred to the general medical floor, and discharged after that in stable medical condition on aspirin, clopidogrel, carvedilol, lisinopril, and atorvastatin.

Case 2

A 29-year-old gravida 2, para 2 African American female recently had a baby by vaginal delivery. On postpartum day 1, she developed acute chest pain. It was pressure like, intermittent, centrally located. and radiated to her left arm and jaw; its intensity increased with movement and relieved gradually with time. The patient reported similar chest pain five years ago when she delivered her first pregnancy; pulmonary embolism (PE) was suspected but CT of thorax was negative for PE and the patient was discharged on aspirin with no complications. Since that time, the patient was pain free till her second pregnancy. The patient said this time, the pain was much worse comparing to her previous pregnancy. Physical exam revealed stable vital signs: blood pressure: 108/68 mmHg, heart rate: 92 beats/min, respiratory rate: 18 breaths/min, temperature: 37.1 degrees Celsius, SpO2: 97% on room air. Cardiovascular examination revealed a pulse with regular rate and rhythm, normal S1 and S2, with no murmurs or added sounds. Respiratory examination was normal with normal vesicular breath sounds heard bilaterally. The rest of physical examination was unremarkable. Laboratory work up was negative except for iron deficiency anemia and hemoglobin was stable in a range of 9-10 g/dL. ECG shows normal sinus rhythm, high sensitivity troponin was elevated at 115 ng/L then dropped to 82 ng/L then to 29 ng/L (normal range 0-0.4 ng/mL); a CT scan for PE was also done this time and was negative.

**Figure 2:**
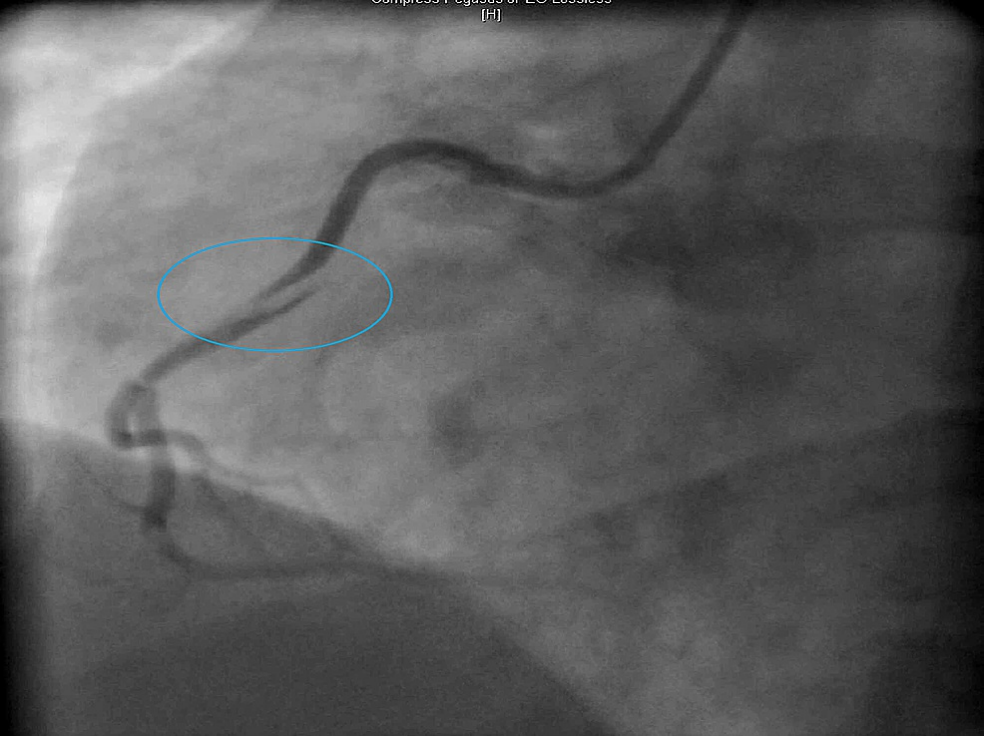
LHC shows spiral dissection of the RCA. LHC, left heart catheterization; RCA, right coronary artery

Due to concern of her severe chest pain and elevated troponin, the patient was started initially on IV heparin infusion, aspirin, and atorvastatin. Left cardiac catheterization was performed and revealed a fresh spiral dissection of the right coronary artery (RCA) as presented in Figure 2 and left coronary dissection likely remote, dating back to her prior pregnancy.

Echocardiogram showed normal left ventricular size, wall thickness, and systolic function. No regional wall motion abnormalities observed. The patient was observed and monitored closely in the MICU for two days and started on dual antiplatelet therapy (DAPT). Given the improvement of symptoms and lack of clear guidelines, percutaneous coronary intervention was not performed. For the foreseeable future, the patient was advised to avoid future pregnancy, oral contraceptives, lifting weights > 10 lbs, and strenuous physical exertion/activity. The patient was discharged on aspirin, metoprolol, and atorvastatin.

Case 3

A 37-year-old African American female patient morbidly obese with no other significant medical history, presented to the ED complaining of chest pain. She described her chest pain as pressure like and tightness in the middle of chest, 7 out of 10 in severity, lasting for five to six minutes then gradually improving and lingering on for some more minutes before completely wearing off. Pain radiated to her back and was not related to activity. The patient denied shortness of breath, nausea, and vomiting. She denied history of heart disease, previous similar chest pain or substance abuse, and she stated that she is quite healthy otherwise.

Physical exam revealed stable vital signs -- blood pressure: 123/78 mmHg, heart rate: 73 beats/min, respiratory rate: 18 breaths/min, temperature: 36.5 degrees Celsius, SpO2: 97% on room air. Cardiovascular examination revealed a pulse with regular rate and rhythm, normal S1 and S2, with no murmurs or added sounds. Respiratory examination was normal with normal vesicular breath sounds heard bilaterally. The rest of physical examination was unremarkable. Urine toxicology and pregnancy test were negative, other laboratory tests were within normal limits.

**Figure 3:**
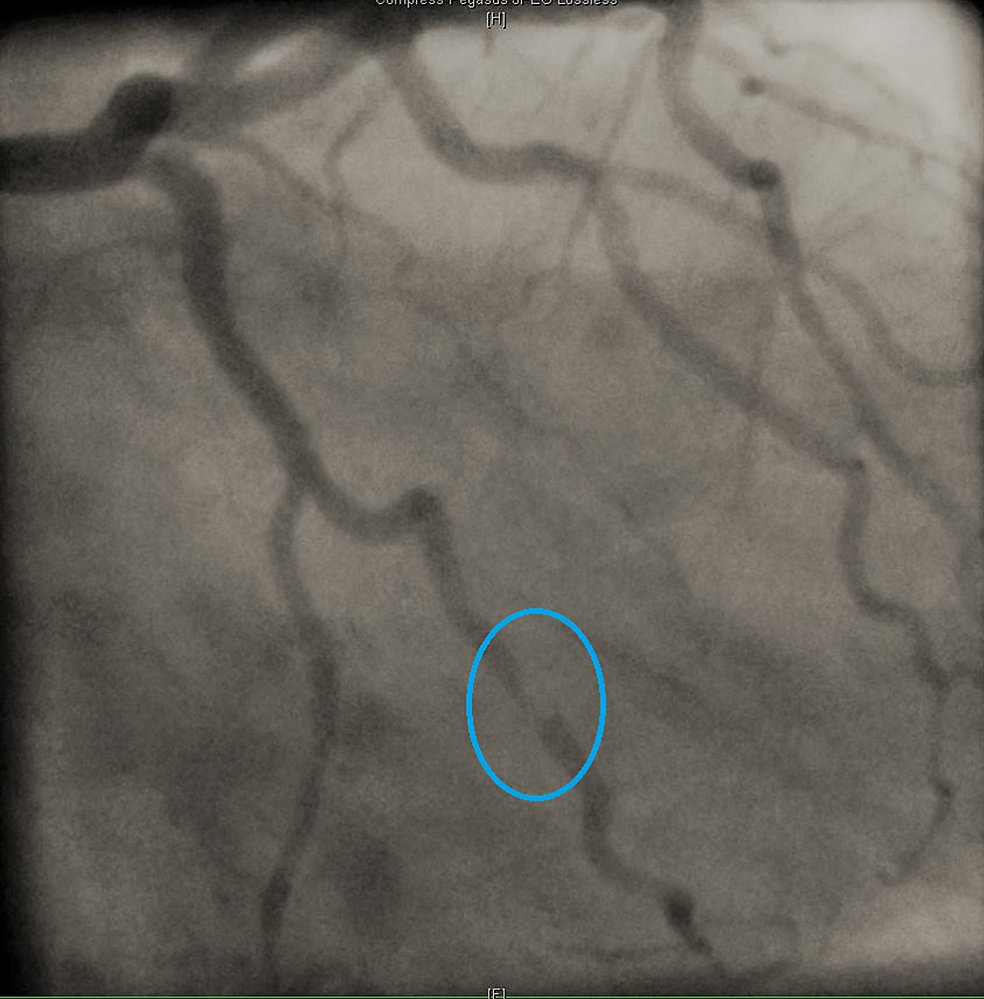
LHC: Type A dissection in obtuse marginal artery as indicated by red arrow. LHC, left heart catheterization

The EKG showed normal sinus rhythm with no ischemic changes, serial high sensitivity troponin was elevated at 440>606>872 ng/mL (normal range 0-0.4 ng/mL). Due to concern of aortic dissection and PE, computed tomography angiography (CTA) and CT PE were performed and both were negative. Transthoracic echocardiogram (TTE) showed normal left ventricular ejection fraction and no regional wall motion abnormalities. Due to her significant chest pain and up trending of troponin, the patient had a LHC. LHC showed 70% stenosis of the first obtuse with type A dissection (Figure 3).

**Figure 4:**
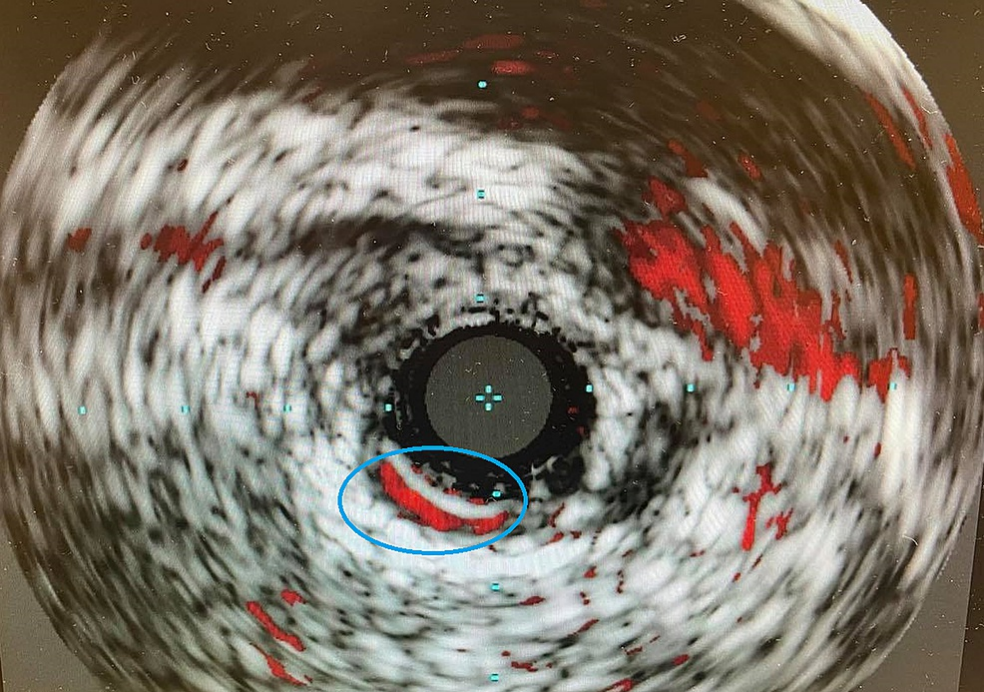
IVUS shows type A spontaneous coronary dissection. IVUS, intravascular ultrasound

Intravascular ultrasound (IVUS) showed type A spontaneous coronary dissection with no significant compromise in lumen as shown in Figure 4. The patient was monitored in the cardiac telemetry unit for one day, she was medically stable, and her chest pain resolved. The patient was discharged on aspirin, metoprolol, and atorvastatin.

## Discussion

Spontaneous coronary artery dissection is caused by sudden splitting of the coronary artery wall not related to atherosclerosis, trauma or iatrogenic causes, resulting in separation of the vessel layers. Possible suggested mechanism is intimal tear or bleeding from the small blood vessels (vasa vasorum) that result in creation of a false lumen filled with intramural hematoma (IMH) [3]. Expansion of the false lumen by an enlarging hematoma may lead to myocardial ischemia and infarction.

Spontaneous coronary artery dissection should be suspected in any young healthy patient, with no risk factors for atherosclerosis like diabetes, hypertension, hyperlipidemia, smoking, etc., who presented with an ACS or cardiac arrest. Risk factors for SCAD include recent childbirth, cocaine abuse, severe high blood pressure, fibromuscular dysplasia, inherited connective tissue diseases such as vascular Ehlers-Danlos syndrome and Marfan syndrome [3]. Idiopathic cases have been reported like our third patient who had no previously mentioned risk factors.

Spontaneous coronary artery dissection occurs most commonly in the third trimester of pregnancy and in the early postpartum period; a recent analysis of a US administrative database found a prevalence of 1.81 SCAD events per 100 000 pregnancies during pregnancy or in the six-week postpartum period [4]. Pregnancy-associated SCAD is more commonly reported in black race; also in patients with history of chronic hypertension, lipid abnormalities, chronic depression, and migraines [4-5].

The etiology of SCAD in pregnancy is still unclear, suggesting precipitating factors are hormonal changes during pregnancy and hemodynamic stress. The estrogen and progesterone receptors present in the coronary arteries may cause changes that weaken the vessel wall leading to arterial wall rupture and development of IMH [1]. Formation of an IMH causes coronary artery obstruction which induces myocardial injury.

When compared with nonpregnant women with SCAD, pregnancy-associated SCAD is associated with a higher incidence of multi-vessel dissections resulting in significant myocardial injury and with higher incidence of cardiogenic shock, arrhythmias, emergent coronary artery bypass surgery and a high maternal and fetal mortality [6]. An accurate diagnosis is needed in order for these women to be advised about future pregnancy, as the risk of recurrent SCAD is remarkable. Coronary angiography is the recommended procedure to make the diagnosis of SCAD, in patients for whom the diagnosis is unclear with coronary angiography, intracoronary imaging with optical coherence tomography (OCT) or intravascular ultrasound (IVUS) may be helpful [7].

Three types of SCAD were angiographically determined: type 1 is diagnosed when there is evidence of dye staining within the arterial wall with multiple radiolucent lumina visualized on angiography; type 2 SCAD is characterized by diffuse long and smooth stenosis that can vary in severity from mild stenosis to complete occlusion; type 3 SCAD is described as a focal tubular stenosis that mimics atherosclerosis and requires intracoronary imaging for definitive diagnosis [8].

There are no consensus guidelines on management of SCAD. Treatment options include medical management, percutaneous coronary intervention (PCI), or coronary artery bypass grafting (CABG), the choice is primarily dependent on the clinical presentation and the severity of coronary flow occlusion on the angiographic study [9]. Dual antiplatelet agents, heparin and beta-blockers maybe used initially for SCAD to preserve patency of the true lumen and prevent thrombotic occlusion. However, these agents could delay healing of the IMH and lead to dissection extension. Thrombolytic agents should not be used due to an increased risk of bleeding and possible extension of IMH [3].

A conservative approach to acute management of SCAD is preferred; dual antiplatelet therapy and beta-blockers are generally accepted treatment with the potential to reduce arterial shear stress, facilitate healing, and reduce long-term recurrence. Revascularization is reserved only for cases with high risk anatomy like left main coronary artery or left main coronary artery equivalent dissection or hemodynamic instability [7] because PCI has a high rate of technical failure and lack of protective effect against future SCAD events [10]. CABG can be performed when multiple coronary vessels or the left main coronary artery is involved.

Unfortunately, the risk of SCAD recurrence is high. At three years, the risk of recurrent SCAD is around 10% [11]. As our second case, she probably had initial coronary artery dissection in her first pregnancy based on finding on coronary angiography, then presented with recurrent in her second pregnancy.

## Conclusions

This case series highlights the need to raise awareness about SCAD, to facilitate accurate diagnosis promptly, particularly when risk factors for coronary artery disease are absent. Postpartum coronary artery dissection is an important cause of acute MI in young female and pregnant women are at a higher risk. The risk of recurrent SCAD in women who become pregnant after an initial SCAD is notable and can be devastating, so careful re-evaluation of etiology with an emphasis on accurately identifying SCAD in order for these women to make an informed decision before subsequent pregnancies is imperative.
